# Prognostic value of impaired estimated glomerular filtration rate in intravesical BCG-treated non–muscle-invasive bladder cancer patients

**DOI:** 10.1038/s41598-017-01532-7

**Published:** 2017-05-03

**Authors:** Bum Sik Tae, Jung Kwon Kim, Minyong Kang, Chang Wook Jeong, Cheol Kwak, Hyeon Hoe Kim, Ja Hyeon Ku

**Affiliations:** Department of Urology, Seoul National University Hospital, Seoul, Republic of Korea

## Abstract

To evaluate the influence of patient-associated parameters and comorbities, with a special focus on renal function after intravesical adjuvant bacillus Calmette-Gue´rin (BCG) immunotherapy in patients with non–muscle-invasive bladder cancer (NMIBC). We retrospectively reviewed the medical records of patients treated from October, 1991 to December, 2013 at Seoul National University who were diagnosed with NMIBC and treated with intravesical BCG. A total of 344 patients who were diagnosed with NMIBC and treated with intravesical BCG were enrolled in this study. Tumor recurrence was observed in 171 patients (49.3%); progression to higher pT category or grade was found in 68 patients (15.1%). Multivariate analysis demonstrated that recurrent tumors and the presence of multiple tumors increased the risk of recurrence. However, other factors also appeared to predict recurrence, such as impaired renal function (<60 ml/min), which was associated with recurrence in univariate and multivariate analyses (HR 1.879 p = 0.008). It is worthy of notice that impaired renal function was an independent predictor of tumor recurrence after BCG instillation in multivariate analysis. Therefore, we should consider not only the clinical or pathologic findings of a tumor but also renal function during decision-making for additional therapy.

## Introduction

Approximately 80% of all newly-diagnosed bladder cancers are found to be non–muscle invasive^1, 2^. Non–muscle-invasive bladder cancer (NMIBC) is defined as a tumor that invades up to the lamina propria, but not into the detrusor muscle. Initial treatment for NMIBC involves transurethral resection of the bladder tumor (TURBT) to remove all visible pathology. Further therapy is determined by pathologic stage and grade of the cystoscopically obtained bladder tumor.

Treatment strategies for intravesical therapy of non–muscle-invasive bladder cancer (NMIBC) have not changed significantly over the past three decades. Patients with high-grade Ta and T1 or CIS NMIBC are at a high risk for recurrence and, more importantly, progression. Because of these findings, both the AUA and EUA recommend initial intravesical treatment with bacillus Calmette-Guerin (BCG) followed by maintenance therapy for a minimum of 1 year^3, 4^. The complete response rate to BCG therapy in patients with high-risk NMIBC can be as high as 83.8%; however, most patients with high-risk disease suffer from recurrence^5^. It has been estimated that as many as 50% of patients with high-risk disease will experience recurrence within 1 year and 90% will do so within 5 years^6^.

Prognostic factors of NMIBC have been the subject of numerous publications over many years^7^. A tool to predict the response to intravesical immunotherapy would be invaluable because early cystectomy may save some non-responders to BCG therapy^8^. In CUETO trials, authors concluded that female gender, history of recurrence, multiplicity, and presence of associated CIS are significant independent predictors for recurrence after BCG instillation^9^.

However most studies have tended to focus on the clinical or pathologic findings of the tumor. Numerous departmental studies evaluated the prognostic usefulness of test parameters from pretreatment complete blood count (CBC) or other laboratory findings for predicting outcomes in cancer patients^10, 11^. Recently estimated glomerular filtration rate (eGFR) has emerged as a prognostic factor for NMIBC recurrence^12, 13^. So we hypothesized that certain preoperative laboratory findings would be associated with unfavorable outcomes of BCG treatment for NMIBC.

## Materials and Methods

### Ethics statements

The Institutional Review Board of Seoul National University Hospital approved this study (H-1609-012-789). Because we retrospectively performed our investigation, the IRB waived the need for informed consent documents from our patients. Patient information was anonymized and de-identified before we carried out the study. All study procedures were carried out in accordance with the Declaration of Helsinki guidelines.

### Study samples

We retrospectively evaluated data collected from 344 patients who underwent TURBT and were treated with BCG instillation between October, 1991 and December, 2013 in our department.

### Study design

Medical records were reviewed for tumor category and grade, presence of comorbidities, and putative preoperative risk factors (Hemoglobin, C-reactive protein (CRP) level, eGFR, age at diagnosis, and gender). Patients with visible tumors underwent complete transurethral bladder resection and were staged according to the 1987 TNM classification and the World Health Organization 1973 grading system. Follow-up of patients was performed in an outpatient setting according to the contemporary guidelines (European Association of Urology Guidelines). Serum samples for evaluation were drawn on the day before the operation. eGFR was calculated using the CKD-epidemiology collaboration formula, with an eGFR of <60 ml/min as the threshold value for impaired renal function. Hb values were stratified into either normal or anemic based on a cut-off value of 13 g/dL in male and 12 g/dL in female patients, as determined by the World Health Organization (Beutler and Waalen, 2006). Recurrence was defined as tumor recurrence with or without pathological upstaging or upgrading. When a patient showed pathological progression by either upstaging or upgrading, tumor progression was recorded. Progression to muscle-invasive disease was defined as the occurrence of a tumor stage greater than or equal to pT2. All statistical analysis was conducted using SPSS® Statistics 21.0. Logistic regression analysis and Chi square tests were performed to assess individual risk factors. Factors associated with the dependent variable at a value of p < 0.05 were included in the multivariate Cox regression model. Kaplan-Meier survival analysis was used to evaluate and illustrate cancer recurrence. The p value was considered statistically significant if it was less than 0.05.

## Results

### Baseline characteristics of the study subjects

A total of 344 patients were eligible for the study. All patients were treated with BCG after TUR of a stage Ta/T1 tumor. Seventy-two patients underwent maintenance BCG using the SWOG protocol, with a median of three courses given (3 weekly instillations per course, range 1–6)^5^. Four patients showed adverse events (fever), and two of them developed sepsis. Among them, one patient who developed pneumonia died because of uncontrollable sepsis. The median follow-up period was 57.2 months. Patient age at diagnosis ranged from 27 to 91 years, with a median age of 64 years. Of all patients, 58 (16.9%) were women. The main characteristics of the patients are given in Table 1.

**Table 1:** Clinical and pathological characteristics of patients.

Parameter	No (%)
Age, Yr	
<60	105 (30.5%)
61–70	125 (36.3%)
>70	114 (33.1%)
HTN	134 (38.5%)
DM	69 (19.8%)
Gender	
Male	286 (83.1%)
Female	58 (16.9%)
No. of tumors	
1	128 (37.2%)
2–7	181 (52.6%)
7<	35 (10.2%)
Tumor size	
<3Cm	248 (72.1%)
>3Cm	96 (27.9%)
T category	
Ta	67 (19.5%)
T1	272 (79.1%)
Associated CIS	
No	262 (76.2%)
Yes	82 (23.8%)
eGFR	
<60ml/min	151 (43.9%)
>60 ml/min	176 (51.2%)
BCG	
Initial instillation 6>	21 (6.0%)
BCG maintenance	72 (20.7%)

In 151 cases, impaired renal function (eGFR < 60 ml/min) was observed. No patient required dialysis or had a history of renal transplantation. No patient had bilateral upper tract dilatation. Impaired eGFR due to a solitary kidney following nephrectomy was observed in 17 patients (16 upper tract TCC and 1 renal cell carcinoma patient).

### Association between parameters and recurrence after BCG treatment

**Figure 1:**
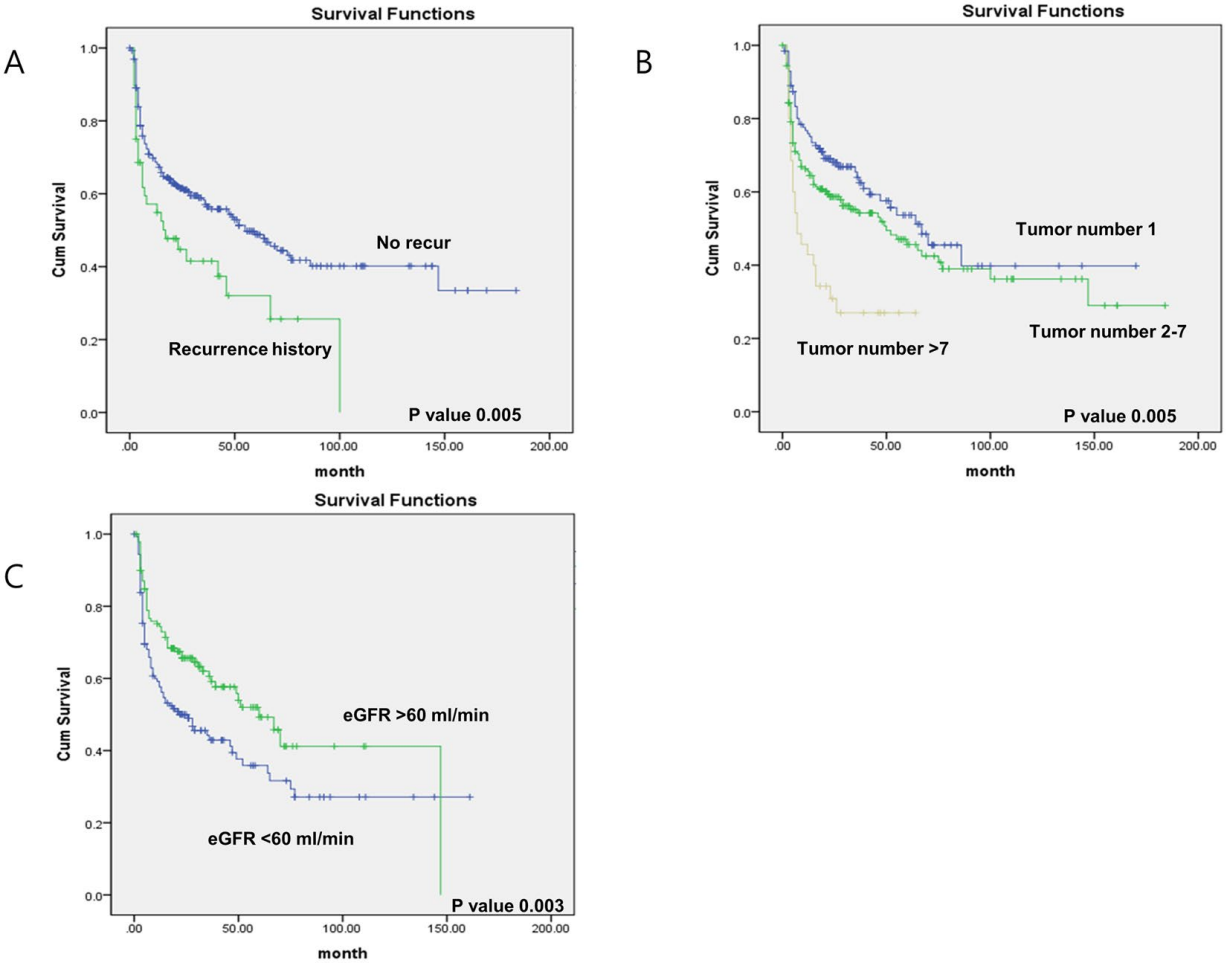
Probability of bladder cancer recurrence-free survival in 344 patients treated with intravesical BCG instillation for NMIBC; Stratified by predictors for recurrence. History of recur (**A**), Tumor number (**B**), Renal function (**C**).

Tumor recurrence was observed in 171 patients (49.7%). There were significant differences in baseline characteristics between the recurrent group and the non-recurrent group concerning tumor grade, multiplicity, previous recurrence history, and eGFR (Table 2). The results of univariate and multivariate testing are shown in Table 3. Univariate analysis revealed number of tumors (tumor number 2–7, HR = 1.859, p = 0.010; tumor number >7, HR = 4.057, p = 0.004), previous recurrence history (HR = 2.062, p = 0.033), previous upper urinary tract tumor history (HR = 5.768, p = 0.002), and impaired renal function (2.481, p = 0.004) as significant predictive determinants for recurrence. Age, gender, anemia, T stage, grade, and CRP level elevation were not significant predictors in the univariate analysis. Variables that represented prior recurrence history, impaired renal function, and number of tumors were included in the final multivariate models for time to recurrence. Previous upper urinary tract tumor history was not included in the final models. Figure 1 shows Kaplan-Meier distributions of time to first recurrence for those variables that were significant in the multivariate analysis. Overall 2-year recurrence-free survival (RFS) rates in the impaired renal function patients were poor compared to those in the normal renal function group (50.0% vs 67.2%, p = 0.003). The 2-year recurrence-free survival rates were 68.0% in the single tumor group, 58.7% in the group with 2–7 tumors, and 30.9% in the group with >7 tumors (p = 0.005). Also, patients who had a history of recurrence showed reduced RFS compared to those with no history of recurrence (2 yrs; 44.7% vs 61.5%, p = 0.005).

**Table 2:** Patient and tumor characteristics of recurrence and non-recurrence groups.

Parameter	No recur (n = 171)	Recur (n = 173)	P
Gender (Female)	25 (14.6%)	30 (17.3%)	0.294
Age			0.631
<60	50 (29.2%)	55 (31.8%)	
61–70	65 (38.0%)	60 (34.7%)	
70<	61 (35.7%)	52 (30.1%)	
Anemia	35 (20.4%)	39 (22.5%)	0.203
Impaired renal function (<60 ml/min)	64 (37.4%)	87 (50.3%)	0.001
Previous UUT History	3 (1.8%)	12 (6.9%)	0.010
Prev. Recur History	17 (9.9%)	32 (18.5%)	0.017
T stage			0.570
Ta	29 (17.0%)	38 (20.0%)	
T1	120 (70.2%)	114 (65.9%)	
CIS	19 (11.1%)	20 (11.6%)	
Grade			0.038
Unknown	0 (0%)	3 (1.7%)	
Low grade	18 (10.5%)	30 (17.3%)	
High grade	153 (89.5%)	140 (80.9%)	
Concomitant CIS	45 (26.3%)	37 (21.4%)	0.172
Size			0.219
<3Cm	127 (74.3%)	121 (69.9%)	
≥3Cm	44 (25.7%)	52 (30.1%)	
Tumor Number			0.003
1	74 (43.3%)	54 (31.2%)	
2–7	88 (51.5%)	93 (53.8%)	
7<	9 (5.3%)	26 (15.0%)	
CRP	76.78 ± 18.91	69.37 ± 16.30	0.601

**Table 3:** Univariate and multivariate analyses of predictors for recurrence after intravesical BCG treatment.

Parameter	Univariate		Multivariate	
	HR	P value	HR	P value
Age				
<60	Ref	Ref	—	—
61–70	0.685 (0.305–1.539)	0.360	—	—
70<	0.479 (0.207–1.109)	0.086	—	—
Gender (Female)	1.799 (0.931–3.473)	0.080	—	—
Tumor Number				
1	Ref	Ref	Ref	Ref
2–7	1.849 (1.056–3.238)	0.031	1.786 (1.067–2.988)	0.027
7<	4.057 (1.576–10.443)	0.004	2.441 (1.209–4.930)	0.013
Tumor size >3Cm	1.588 (0.820–3.077)	0.170	—	—
Grade : LG/HG	2.273 (0.924–6.161)	0.072	—	—
Associated CIS	0.899 (0.491–1.644)	0.729	—	—
CRP	0.877 (0.663–1.159)	0.357	—	—
Anemia	0.837 (0.762–1.023)	0.098	—	—
Impaired renal function (<60 ml/min)	2.481 (1.331–4.628)	0.004	1.879 (1.178–2.999)	0.008
Prev. Recur History	2.062 (1.059–4.015)	0.033	1.651 (1.102–2.475)	0.015
Previous UUT	5.768 (1.936–17.183)	0.002	2.002 (0.981–3.905)	0.053
T category	2.787 (0.814–9.545)	0.103	—	—
Initial instillation 6>	1.815 (0.612–5.379)	0.282	—	—

### Association between parameters and progression after BCG treatment

Tumor progression to a higher pathologic T category or grade was observed in 68 (19.7%) patients (median time to progression, 60.1 months), and 52 (15.1%) patients underwent radical cystectomy. In the univariate analysis, only tumor grade (low vs. high) was a significant prognostic factor for tumor progression, with an HR of 2.50 (95% CI: 1.09–5.73, p = 0.03). However, this factor did not reach significance in the multivariate analysis (Table 4).

**Table 4:** Univariate and multivariate analyses of predictors for progression after intravesical BCG treatment.

Parameter	Univariate		Multivariate	
	HR	P value	HR	P value
Age				
<60	Ref	Ref	—	—
61–70	0.919 (0.355–2.380)	0.861	—	—
70<	1.078 (0.420–2.768)	0.876	—	—
Gender (Female)	0.311 (0.041–2.383)	0.261	—	—
Tumor Number				
1	Ref	Ref	Ref	Ref
2–7	1.931 (0.562–6.508)	0.299	1.710 (0.734–3.981)	0.214
7<	4.451 (0.793–24.971)	0.090	2.169 (0.643–7.313)	0.212
Tumor size <3Cm	0.460 (0.125–1.692)	0.243	—	—
Grade : LG/HG	2.50 (1.09–5.73)	0.03	4.189 (0.417–42.107)	0.224
Associated CIS	0.377 (0.065–1.348)	0.116	—	—
Anemia	0.496 (0.140–1.753)	0.276	—	—
CRP	1.222 (0.928–1.609)	0.153	—	—
Impaired renal function (<60 ml/min)	0.656 (0.264–2.312)	0.656	1.431 (0.765–2.678)	0.262
Prev. Recur History	2.057 (0.787–5.379)	0.141	2.076 (0.842–5.118)	0.112
Previous UUT	2.464 (0.859–7.072)	0.094	2.304 (0.817–6.495)	0.115
T category	2.437 (0.511–11.614)	0.264	—	—
Initial instillation 6>	2.767 (0.353–21.717)	0.333	—	—

## Discussion

Attempts at identification of prognostic factors that predict recurrence and progression for patients with non–muscle-invasive bladder cancer have been undertaken by a variety of groups. In particular, randomized phase 3 studies (CUETO trials) presented prognostic factors in patients treated only with BCG^9^. In the CUETO trials, the authors presented significant independent predictors for recurrence as female gender, history of recurrence, multiplicity, and presence of associated CIS. However, there was no evaluation of the correlation between impaired renal function and NMIBC recurrence after BCG treatment.

In our study, aside from the established pathological risk factors, history of recurrence and multiplicity, impaired renal function was a strong independent predictor of tumor recurrence after BCG treatment in univariate and multivariate analyses. However, it should be noted that our model does not include the risk factors of CIS and female gender that were found to be significantly predictive in the CUETO trials. But, it is notable that impaired renal function was a strong risk factor for recurrence following BCG treatment. To our knowledge, this is the first study on the correlation between renal function and NMIBC recurrence after BCG treatment.

Impaired renal function, which is common in cancer patients, is considered to be a relevant clinical parameter for bladder cancer^14^. Some studies demonstrated that impaired renal function is correlated with recurrence and progression of NMIBC. In a retrospective study by Rausch S. *et al*., the authors found that impaired eGFR is a strong independent predictor of not only tumor recurrence but also tumor progression^12^. In addition, Jian Cao *et al*. demonstrated that patients with renal insufficiency exhibit a higher risk of bladder cancer recurrence, which may be because of an increased prevalence of immunosuppressive conditions in these patients^15^.

In this study, which involved 344 patients with NMIBC who received transurethral resection (TUR) and BCG treatment, impaired renal function, previous recurrence history, and tumor multiplicity were identified as independent risk factors for disease recurrence. Some previous studies demonstrated that renal insufficiency was common in patients with NMIBC. For example, Cao J. *et al*. found that 26.3% of NMIBC patients had renal insufficiency^15^. Furthermore, 151 of the patients in our study (43.9%) had renal insufficiency, with an eGFR of ≤60 ml/min/1.73 m^2^. However, this was a single-center report from a top academic hospital in Korea, which usually receives a greater proportion of cancer patients with complicated and severe diseases, possibly accounting for the substantially higher rate of patients with impaired renal function in our study.

There are some hypotheses that may explain recurrence of NMIBC after BCG treatment in patients with impaired renal function. First, it may be associated with immune function. T helper type 1 (Th1) lymphocytes secrete interleukin (IL)-2, interferon-γ, and lymphotoxin-α. Th1 lymphocytes stimulate type 1 immunity, which is characterized by intense phagocytic activity. Conversely, T helper type 2 (Th2) cells secrete IL-4, IL-5, IL-9, IL-10, and IL-13. Th2 lymphocytes stimulate type 2 immunity, which is characterized by high antibody titres^16^. There is strong evidence that the success of BCG treatment might be due to a preferential induction of a Th-1 response (detected in the urine by analysis of relevant cytokines)^17, 18^. Although somewhat controversial, Th-2 responses detected in the bladder are associated with poorer outcomes and might explain failure to respond to BCG treatment^19^. Recently, several authors have reported that uremic patients exhibit a change in the profile of Th-1/Th-2 lymphocytes, which would explain, at least in part, the immunological abnormalities found in these patients. Especially, predominance of Th-2 over Th-1 is seen in CKD patients, which suggests an increase in the rate of Th-1 lymphocyte apoptosis relative to that of Th-2^20^. So, we assume that increased Th-2 responses in CKD patients may influence poor outcomes following BCG treatment.

Another possible explanation for the relevance of renal insufficiency for recurrence of NMIBC after BCG treatment is differences in urine composition between individuals with and without CKD. Albuminuria is a feature of CKD that is known to indicate end-stage renal disease, cardiovascular morbidity and mortality, and cancer. Roth *et al*. analyzed urine samples from patients with CKD and found an increased level of cytokeratin 18 in comparison with samples from healthy controls as a surrogate for increased inflammation and consecutive epitheloid necrosis and apoptosis^21^. It may be that elevated oncogenic activity of inflammatory proteins in the urine of patients with CKD contributes to an earlier recurrence in patients with renal impairment. So, we assume that this can influence recurrence in NMIBC patients treated with BCG.

It is known that diabetes and hypertension are the leading causes of chronic kidney disease in elderly patients; however, an exact diagnosis is often difficult. Diabetes especially was shown to be associated with a modestly increased risk of bladder cancer in a meta-analysis^22^. However, in the present study, not all patients with renal impairment had a clear diagnosis of the cause, especially those with moderately impaired eGFR. And, the presence of diabetes did not reach significance in univariate and multivariate analyses in this study. Moreover, considering the complex etiology of renal impairment, it is unlikely that a study with the current sample size has enough power to detect an association between a specific cause of renal impairment and NMIBC recurrence after BCG treatment.

Previous upper urinary tract history exhibited statistical significance in univariate analysis. However, it failed to reach significance in our multivariate model. We suspect that this is due to the small number of patients whose records contained UUT history (n = 15, 4.4% of our total study population). Despite the small sample size, previous UUT history showed a correlation with recurrence after BCG treatment in NMIBC. This result suggests that a more compelling difference may be observed in a study with a larger sample size.

In previous studies, several laboratory findings obtained from blood tests, including C-RP^23^ and hemoglobin levels^13^, have been found to be associated with the treatment outcomes of patients with bladder cancer. However, there was no significant association with recurrence after BCG treatment in our study.

Some studies have shown that CRP is a prognostic factor in metastatic bladder cancer and MIBC^23, 24^. Hendrik Eggers *et al*. evaluated the prognostic role of pretreatment serum C-RP levels in a retrospective study of 34 patients. The authors revealed that a C-RP level ≥80 mg/l is an independent risk factor for poor overall survival^23^. Yoshida *et al*. defined an elevated CRP level as >0.5 mg/dl and found that patients with a CRP level above the cut-off have a significantly shorter cancer-specific survival time than do their counterparts^24^. However, we did not find a significant association between elevated CRP levels and NMIBC recurrence after BCG treatment. This result may be because the NMIBC is mainly limited to the mucosal layer of the bladder and thus has a minimal influence on systemic inflammatory status^12^.

Other authors demonstrated that anemia is a prognostic factor in NMIBC. Orcun *et al*. evaluated the prognostic role of preoperative anemia on long-term survival outcomes in NMIBC^25^. They found that 118 (36.9%) patients with anemia showed decreased overall survival. However, there have been few studies on the correlation between NMIBC recurrence and anemia. Also, there was no significant association found between anemia and recurrence after BCG treatment in our study.

There were some limitations to our study. First, it is limited by its retrospective design. We could not conduct correlation analysis of relevant cytokines in CKD patients with tumor recurrence after BCG treatment. Secondly, the small sample size, short follow-up time, and low mortality rate in our study prohibited us from analyzing impaired renal function as a survival factor. Therefore, a large prospective study to assess the role of renal impairment in bladder BCG instillation therapy can help to confirm the results of our analysis.

In conclusion, our study delivers important information to both NMIBC patients and their health care providers regarding methods of predicting the prognosis of NMIBC and postoperative management of the disease. Since impaired renal function is a risk factor for NMIBC recurrence after BCG treatment, we need to more closely monitor patients with renal insufficiency.

## Conclusion

Impaired renal function was revealed to be an independent predictor of recurrence of tumor after BCG instillation in univariate and multivariate analyses. So, we should consider not only clinical or pathologic findings of tumors but also renal function of patients during counseling and decision-making for additional therapy.
